# Effects of chronic high-altitude exposure on memory function in indigenous highland junior high school students: behavioral and electroencephalographic evidence

**DOI:** 10.3389/fnins.2026.1719666

**Published:** 2026-03-10

**Authors:** Xintong Chen, Weigang Gong, Xuan Lyu, Huiju Shi, Xiang Li, Qiang Zhu, Chun Zheng, Chao Fu

**Affiliations:** 1 School of Education, Qinghai Normal University, Xining, Qinghai, China; 2 School of Nursing, Qingdao University, Qingdao, China; 3 Department of Traditional Chinese Medicine, Bijie Medical College, Bijie, Guizhou, China; 4 School of Education, Qinghai Minzu University, Xining, Qinghai, China; 5 Qinghai Plateau Brain Science Research Center, Xining, Qinghai, China; 6 Qinghai Cardio-Cerebrovascular Specialty Hospital, Qinghai High Altitude Medical Research Institute, Xining, Qinghai, China

**Keywords:** chronic hypoxia, encoding, high-altitude exposure, indigenous highland junior high school students, maintenance, memory function, retrieval

## Abstract

**Background:**

This study investigated the effects of chronic high-altitude exposure on memory function and neural processing in indigenous highland junior high school students.

**Methods:**

Three altitude groups were established–low (1,400 m), mid (2,800 m), and high (4,200 m). A delayed recognition task dissociated memory encoding, maintenance, and retrieval stages. Physiological (blood oxygen saturation, heart rate, vital capacity), behavioral, and electroencephalographic measures were employed.

**Results:**

Physiological: Blood oxygen saturation, heart rate, and maximal vital capacity decreased with increasing altitude. Behavioral: The high-altitude group showed lower recognition discriminability and more conservative decision-making vs. lower-altitude groups. Electrophysiological: High-altitude subjects exhibited reduced encoding-related attention, altered maintenance activity, and attenuated early retrieval attention. Correlation analyses linked blood oxygen saturation to behavioral discriminability and event-related potential components across memory stages.

**Conclusion:**

Chronic hypoxic exposure associates with stage-specific alterations in memory-related neural and behavioral performance in adolescent highlanders. While more pronounced impairments occurred at 4,200 m, these findings are preliminary; finer-grained altitude sampling and larger samples are needed to identify critical altitude thresholds.

## 1 Introduction

Memory is a core function of the human cognitive system and provides the foundation for learning, reasoning, and adaptive behavior. It encompasses a dynamic psychological process through which individuals encode, maintain, and retrieve external information (Wang et al., 2009). According to information-processing theory, memory consists of three interrelated stages: encoding, maintenance, and retrieval (Girardeau and Lopes-Dos-Santos, 2021). Encoding refers to the transformation of sensory input into neural representations through perceptual and attentional processes. Maintenance involves the temporary storage and consolidation of encoded information through sustained neural activity and synaptic modulation. Retrieval reflects the process of accessing stored information for current cognitive demands and is commonly assessed through recognition or recall performance (Peng, 2023). These stages are functionally interdependent: ineffective encoding limits available information, unstable maintenance compromises retention, and retrieval performance reflects the cumulative quality of preceding processes.

High-altitude environments are characterized by reduced atmospheric pressure and decreased oxygen availability, resulting in chronic hypoxic stress. Sustained hypoxia induces a series of physiological responses, including changes in cerebral perfusion, energy metabolism, neurotransmitter regulation, and vascular function (Luks and Hackett, 2022). Because the brain relies heavily on continuous oxygen supply to support neural activity, prolonged hypoxic exposure may place constraints on cognitive processing efficiency. In particular, memory functions—especially those requiring sustained attention, executive control, and working memory resources—may be vulnerable to chronic reductions in oxygen availability. These considerations raise an important question: does long-term exposure to high-altitude hypoxia influence memory functioning, and if so, through which physiological and neural mechanisms?

Extensive evidence from acute and simulated high-altitude exposure studies indicates that hypoxia can impair multiple cognitive domains, including memory, attention, and executive control (Zhu et al., 2019). For example, individuals acutely exposed to hypobaric hypoxia at 3,000–4,000 m have been shown to exhibit reduced memory accuracy and prolonged reaction times (Wesensten et al., 1993; Yan et al., 2011). However, findings from populations exposed to chronic hypoxia remain inconsistent. Some studies suggest that long-term residents at high altitude exhibit persistent impairments in working memory, response inhibition, and information maintenance (Bakharev, 1981; Ma et al., 2020). In contrast, other investigations have reported comparable cognitive performance between high-altitude natives and lowland controls, attributing such findings to developmental or physiological adaptation during growth (Hogan et al., 2010; Richardson et al., 2011). These divergent results indicate that the cognitive consequences of chronic hypoxia may depend on altitude level, developmental stage, and individual adaptation processes.

Despite important progress, several limitations remain in the existing literature. First, many studies have focused on acute or simulated hypoxia, whereas fewer investigations have examined chronic exposure under natural living conditions. Second, adolescents—who undergo rapid neural and cognitive development—have been relatively underrepresented, limiting developmental interpretations. Third, insufficient integration of physiological, behavioral, and neural measures has hindered comprehensive mechanistic explanations. Fourth, many studies have relied solely on behavioral indices, which provide limited insight into stage-specific neural processes. To address these limitations, the present study investigated indigenous junior high school students residing at three representative altitudes—low altitude, mid altitude, and high altitude—under ecologically valid conditions. By combining physiological indicators, behavioral performance, and electroencephalographic (EEG) measures during a delayed recognition task (Morgan et al., 2008), this study systematically examined memory encoding, maintenance, and retrieval processes. This integrative design enables a multi-level analysis of how chronic hypoxic exposure influences adolescent memory function and its underlying neural dynamics with high temporal resolution.

Based on previous physiological research, chronic hypoxic exposure is expected to influence cardiovascular and respiratory regulation, including blood oxygen saturation (SpO_2_), heart rate, and maximal vital capacity (MVC) (Luks and Hackett, 2022). Accordingly, the present study hypothesized that these physiological indicators would show systematic altitude-related changes. At the behavioral level, extensive evidence suggests that both acute and chronic hypoxia may impair memory accuracy and processing speed (Wesensten et al., 1993; Tao et al., 2024). Therefore, it was hypothesized that increasing altitude would be associated with reduced recognition accuracy and prolonged reaction times. In addition, because semantic processing facilitates memory encoding, better performance was expected for Chinese character stimuli than for picture stimuli (Xiao, 2008).

At the neural level, this study focused on event-related potential (ERP) components associated with distinct memory stages. During encoding, the parietal P3 component reflects attentional resource allocation and stimulus evaluation (Rugg et al., 1998; Zhang et al., 2014). Previous studies have reported reduced P3 amplitudes under chronic hypoxic conditions, suggesting diminished attentional resources (Chiu et al., 2010). Accordingly, we hypothesized that P3 amplitude would decrease with increasing altitude. During maintenance, sustained frontal negativity—commonly referred to as the negative slow wave (NSW)—reflects continuous information rehearsal and working memory load (Ruchkin et al., 1992; Wang et al., 2016). Given evidence that chronic hypoxia impairs response inhibition and information maintenance (Ma et al., 2020), we hypothesized that NSW amplitude would show altitude-related alterations indicative of compromised maintenance processes. During retrieval, the frontal N1 component indexes early perceptual and selective attention processes (Vogel and Luck, 2000). Although long-term residents may exhibit partial adaptation, previous studies suggest that chronic hypoxia may constrain early sensory-attentional processing under high metabolic demand (Barkaszi et al., 2016). Therefore, we hypothesized that N1 amplitude might be attenuated at higher altitudes, reflecting subtle limitations in early perceptual efficiency.

## 2 Materials and methods

### 2.1 Participants

Participants in this study were second-year junior high school students recruited from three regions:

Zhangye City, Gansu Province (altitude: 1,400 m), Menyuan County, Haibei Prefecture (altitude: 2,800 m), and Zhiduo County, Yushu Prefecture (altitude: 4,200 m). These groups are hereafter referred to as the low-altitude (LA), mid-altitude (MA), and high-altitude (HA) groups, respectively. Geographical information and participants’ demographic characteristics are presented in Table 1.

**TABLE 1:** Participant demographic and geographical information.

Source region	Altitude (m)	Oxygen content (%)	Atmospheric pressure (Kpa)	Longitude	Latitude	N	Male	Female	Mean age (years)
Zhangye city	1,400	18.59	84	100	38	26	14	12	13.97
Menyuan county	2,800	16.34	66	101	37	26	12	14	13.85
Zhiduo county	4,200	14.24	56	95	33	25	12	13	14.64

Geographical data (altitude, oxygen content, atmospheric pressure, longitude, and latitude) were obtained from Xinzhi Satellite Map.

All participants were right-handed, in good physical health, had normal or corrected-to-normal vision, and reported no history of color vision deficiency, neurological disorders, or psychiatric illness. This study was approved by the relevant institutional ethics committee and was conducted in accordance with the Declaration of Helsinki. Permission was obtained from participating schools, and written informed consent was obtained from both participants and their legal guardians prior to data collection. Participation was voluntary.

### 2.2 Experimental materials

**FIGURE 1:**
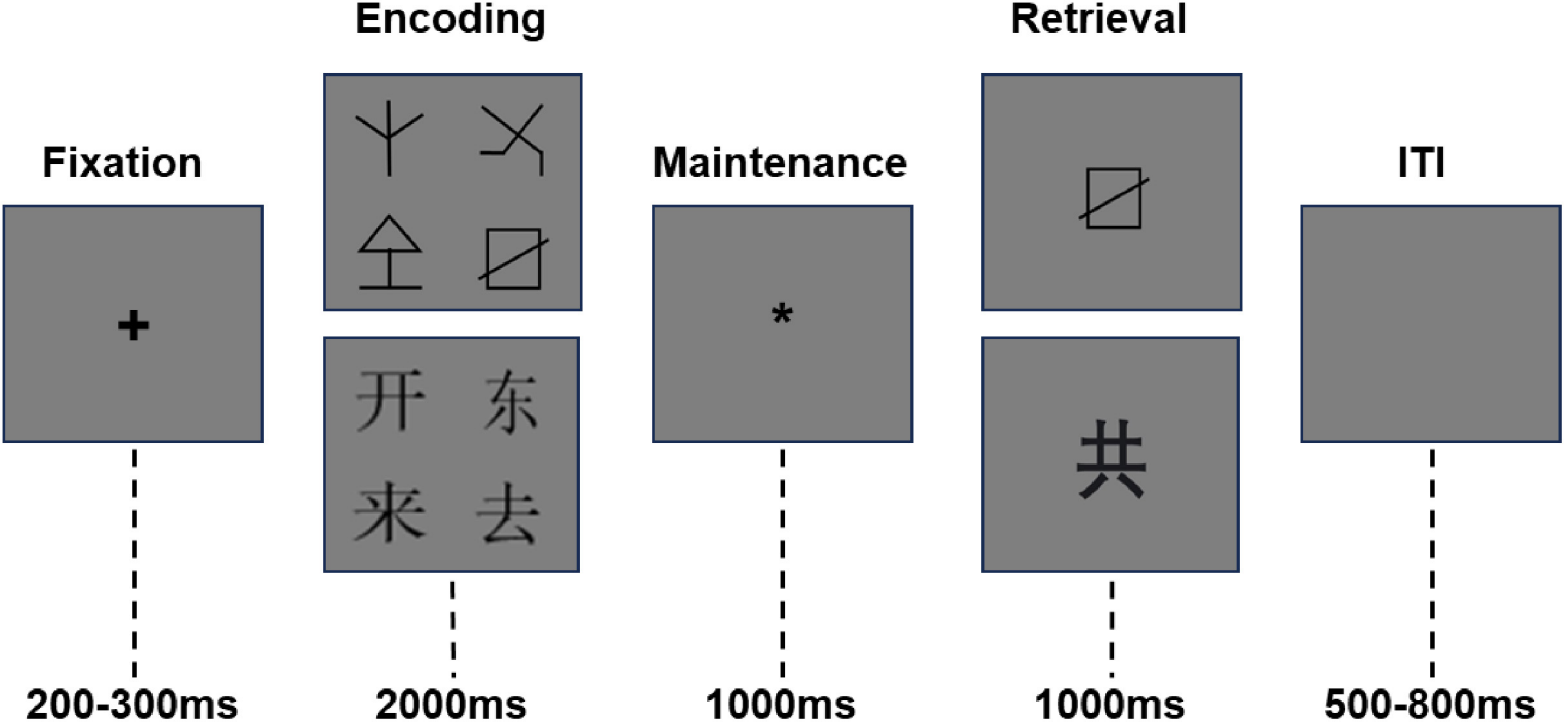
Experimental flowchart. A random inter-stimulus interval (ISI; 500–800 ms) occurred between screens but is not depicted for visual clarity. During the maintenance stage, the asterisk (*) appearing in the center of the screen serves to cue participants to retain the encoded information in working memory.

The experiment employed two types of memory stimuli: pictures and Chinese characters. The picture stimuli consisted of 15 non-linguistic graphic symbols with moderate difficulty and comparable visual complexity (Guo et al., 2003). The Chinese character stimuli comprised 15 commonly used single-character words, each containing 4–7 strokes. Prior to the formal experiment, material difficulty and visual complexity were evaluated by an independent sample of participants who did not take part in the EEG recordings. In each altitude group, 30 students from the same grade but different classes were recruited to rate both picture and Chinese character stimuli on two dimensions: difficulty and visual complexity. Ratings were obtained using a 6-point Likert scale (1 = not at all complex/difficult; 6 = extremely complex/difficult). Mean ratings for both dimensions were approximately 3, indicating moderate and comparable difficulty and complexity. No significant differences were observed across altitude groups (all *p* > 0.05), confirming the suitability of the materials for all participants. Following established procedures (Zhang et al., 2019), each trial randomly selected four items—either pictures or Chinese characters—and displayed them in the four quadrants surrounding the center of the computer screen, forming a stimulus matrix (Figure 1).

### 2.3 Experimental procedure

Stimuli were presented using E-Prime 3.0 software (Psychology Software Tools, Inc., 2016). Participants were seated approximately 50 cm from the display monitor, resulting in a visual angle of 7.9° × 0.8°. The experimental procedure is illustrated in Figure 1. Each trial began with a fixation cross (“ + ”) presented for 200–300 ms to orient participants’ attention and prepare them for the task. This was followed by the encoding phase, during which a matrix of four pictures or Chinese characters was displayed for 2,000 ms. Participants were instructed to memorize the stimuli for subsequent recognition. Next, a maintenance phase lasting 1,000 ms was presented, during which an asterisk (“*”) appeared at the center of the screen to cue participants to retain the encoded information in working memory. This was followed by the retrieval phase, in which a single picture or Chinese character was presented at the center of the screen for 1,000 ms. Participants were required to judge whether the stimulus had appeared during the encoding phase by pressing the “F” key for “yes” and the “J” key for “no.” The inter-stimulus interval (ISI) between successive screens and the inter-trial interval (ITI) were both randomly varied between 500 and 800 ms to reduce temporal predictability and anticipatory responding. The experiment consisted of a practice phase and a formal experimental phase. During the practice phase, participants completed five trials for each stimulus type (pictures and Chinese characters) to ensure task comprehension. The formal phase consisted of 80 trials in total, including 40 picture trials and 40 Chinese character trials. Trials were presented in blocks, and the order of stimulus type was counterbalanced across participants. Within each block, the ratio of old (previously presented) to new (non-presented) stimuli was 1:1, with 20 trials requiring an “F” response and 20 trials requiring a “J” response. Trial order was randomized within each block. Prior to the experimental task, participants completed Raven’s Progressive Matrices to assess general intellectual ability and ensure group comparability. In addition, physiological indices, including SpO_2_, heart rate, and MVC were measured before and after the experimental session.

### 2.4 EEG data acquisition and preprocessing

EEG signals were recorded using a 64-channel electrode cap (Quik-Cap, NeuroScan Inc.) arranged according to the extended International 10–20 system. Data were acquired using a NeuroScan SynAmps2 amplifier and Curry 8 software (Compumedics NeuroScan, Charlotte, NC, United States). During recording, the reference electrode was placed on the right mastoid, and offline data were re-referenced to the average of the left and right mastoids (M1 and M2). Vertical and horizontal electrooculograms (VEOG and HEOG) were simultaneously recorded to monitor ocular activity. Electrode impedances were maintained below 5 kΩ. EEG signals were sampled at 1,000 Hz with an online bandpass filter of 0.05–100 Hz.

Offline preprocessing was performed using the EEGLAB toolbox (version 2024.2) and custom MATLAB (R2023b) scripts. The detailed procedure consisted of the following steps: 1. Data Import: Continuous data were imported from Curry files and converted to EEGLAB format; 2. Channel Location Assignment: Electrode positions were assigned based on the extended 10–20 system using a custom location file; 3. Downsampling: Data were downsampled from 1000 to 500 Hz to reduce computational load; 4. Filtering: A high-pass filter at 0.1 Hz and a low-pass filter at 45 Hz were applied to remove slow drifts and high-frequency noise, respectively. A 50 Hz notch filter (48–52 Hz) was used to suppress line noise. All filters were implemented using a zero-phase shift model (to prevent temporal distortion of neural signals) with a roll-off rate of 24 dB/octave (ensuring sharp frequency cutoffs and effective attenuation of out-of-band noise); 5. Rereferencing: Data were rereferenced from the initial right mastoid reference to the average of the left (M1) and right (M2) mastoids; 6. Independent Component Analysis (ICA): ICA was performed using the extended INFOMAX algorithm. Ocular and muscular artifacts were identified and removed using the Adjust toolbox (with a threshold of 0.9 for “Eye” and “Muscle” components); 7. Non-EEG Channel Removal: Channels not used for EEG analysis (e.g., EOG, EKG, EMG) were removed; 8 Bad Channel and Segment Rejection (Manual): Continuous data were visually inspected to identify and interpolate bad channels (using spherical splines) and exclude bad segments; 9. Epoching and Baseline Correction: Data were segmented into epochs from –200 to 1,000 ms relative to stimulus onset, and a baseline correction (–200 to 0 ms) was applied; 10. Automatic Epoch Rejection: Epochs containing amplitudes exceeding ± 75 μV were automatically rejected; 11. ERP Averaging: Finally, artifact-free epochs were averaged separately for each experimental condition to obtain event-related potentials (ERPs). After this rigorous preprocessing pipeline, the following number of trials per condition (reported as mean ± standard deviation) were retained for final ERP analysis: for picture stimuli—encoding: 20.92 ± 6.37, maintenance: 21.26 ± 6.14, retrieval: 21.23 ± 6.10; for character stimuli—encoding: 26.30 ± 8.00, maintenance: 26.69 ± 7.62, retrieval: 26.66 ± 7.67. These trial counts ensure sufficient signal-to-noise ratio for reliable ERP analysis. In addition, it should be noted that during the preprocessing of EEG data, one participant from the low-altitude group and one from the mid-altitude group were excluded due to poor signal quality and an insufficient number of valid trials remaining after artifact rejection. Therefore, the sample sizes for ERP analysis were 25, 25, and 25 for the low-, mid-, and high-altitude groups, respectively.

### 2.5 Experimental design and data analysis

This study employed a 3 × 2 mixed experimental design, with altitude as a between-subjects factor with three levels (LA group: 1,400 m; MA group: 2,800 m; HA group: 4,200 m) and material type as a within-subjects factor with two levels (pictures and Chinese characters). Physiological indices, including SpO_2_, heart rate, and MVC, were averaged across pre- and post-experiment measurements. For behavioral performance, signal detection theory indices—namely, discriminability (*d’*) and response criterion (*C*)—were used to characterize recognition performance while accounting for false alarm rates and individual response tendencies (Tanner and Swets, 1954; Wixted, 2020). Only trials with valid keypress responses within the response window were included in the signal detection theory analysis. Trials without responses were excluded from both hit rate (HR) and false alarm rate (FAR) calculations. Accordingly, HR was calculated as the proportion of correctly identified old stimuli among all responded old-stimulus trials, and FAR was calculated as the proportion of incorrect “old” responses among all responded new-stimulus trials. This procedure ensured that HR and FAR were based on the actual number of valid responses and avoided biases associated with fixed denominators in the presence of missing responses. The discriminability index was calculated as *d’* = Z(HR) - Z(FAR), and the response criterion was calculated as *C* = -0.5 × [Z(HR) + Z(FAR)], where *Z* denotes the inverse cumulative normal distribution. Higher *d’* values indicate better discriminability between old and new stimuli, whereas C reflects response bias (*C* > 0: conservative; *C* < 0: liberal).

For EEG data, mean amplitudes of three ERP components were analyzed: the parietal P3 during encoding, the frontal NSW during maintenance, and the frontal N1 during retrieval. All statistical analyses were conducted using SPSS Statistics 27.0 (IBM, Somers, United States). Repeated-measures analyses of variance (ANOVAs) were performed on physiological, behavioral, and EEG measures. When the assumption of sphericity was violated, Greenhouse–Geisser corrections were applied. Unless otherwise specified, ANOVA results are reported as mean ± standard error (SE), whereas descriptive statistics are reported as mean ± standard deviation (SD). The significance level was set at *p* < 0.05.

## 3 Results

### 3.1 Results of the standard Raven’s progressive matrices

The results of the standard Raven’s progressive matrices indicated that the mean scores for the low-, mid-, and high-altitude groups were 46.10 ± 5.70, 45.20 ± 5.30, and 44.80 ± 4.90, respectively. A one-way ANOVA revealed no significant difference in Raven’s scores among the three groups, *F*(2, 74) = 0.65, *p* = 0.53, η*_*p*_*^2^ = 0.02, indicating that the participants were homogeneous in terms of general cognitive ability.

### 3.2 Physiological index results

**FIGURE 2:**
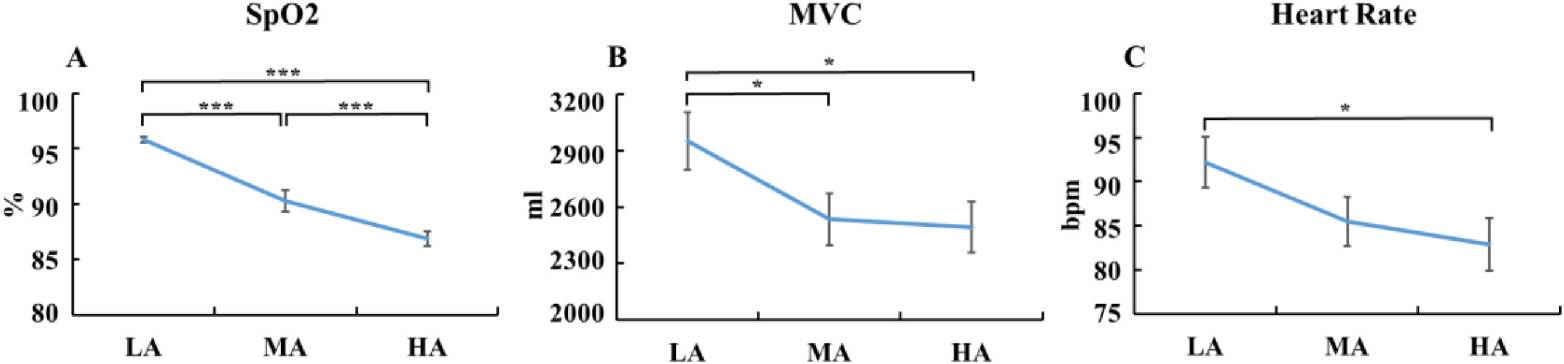
Physiological indices of participants across altitude groups. **(A)** SpO_2_; **(B)** MVC; **(C)** heart rate. SpO_2_, blood oxygen saturation; MVC, maximal vital capacity; LA, low altitude (1,400 m); MA, mid altitude (2,800 m); HA, high altitude (4,200 m). Error bars indicate SE. *p < 0.05, ***p < 0.001.

A one-way ANOVA was conducted to examine the effects of altitude on SpO_2_, MVC, and heart rate. The results (Figure 2) revealed a significant main effect of altitude on SpO_2_, *F*(2, 74) = 41.45, *p* < 0.001, η*_*p*_*^2^ = 0.53. The high-altitude group exhibited significantly lower SpO_2_ (86.88 ± 0.69) compared to the mid-altitude group [90.31 ± 0.96, *p* < 0.001, 95% CI = (1.45, 5.40)] and the low-altitude group [95.81 ± 0.25, *p* < 0.001, 95% CI = (6.95, 10.90)]. Additionally, the mid-altitude group showed significantly lower SpO_2_ than the low-altitude group [90.31 ± 0.96 vs. 95.81 ± 0.25, *p* < 0.001, 95% CI = (3.54, 7.45)]. For MVC, a significant main effect of altitude was found, *F*(2, 74) = 3.16, *p* = 0.048, η*_*p*_*^2^ = 0.08. The low-altitude group exhibited significantly higher MVC (2949.92 ± 153.00) compared to both the mid-altitude group [2534.88 ± 138.67, *p* = 0.042, 95% CI = (15.89, 814.19)] and the high-altitude group [2491.76 ± 134.72, *p* = 0.026, 95% CI = (55.04, 861.29)]. Regarding heart rate, a marginally significant main effect of altitude was found, *F*(2, 74) = 2.65, *p* = 0.077, η*_*p*_*^2^ = 0.07. The low-altitude group had a significantly higher heart rate (92.17 ± 2.91) compared to the high-altitude group [82.88 ± 2.99, *p* = 0.028, 95% CI = (1.01, 17.56)]. No significant differences in heart rate were observed between the low-altitude and mid-altitude groups (92.17 ± 2.91 vs. 85.50 ± 2.82) or between the mid-altitude and high-altitude groups (85.50 ± 2.82 vs. 82.88 ± 2.99).

### 3.3 Behavioral results

**FIGURE 3:**
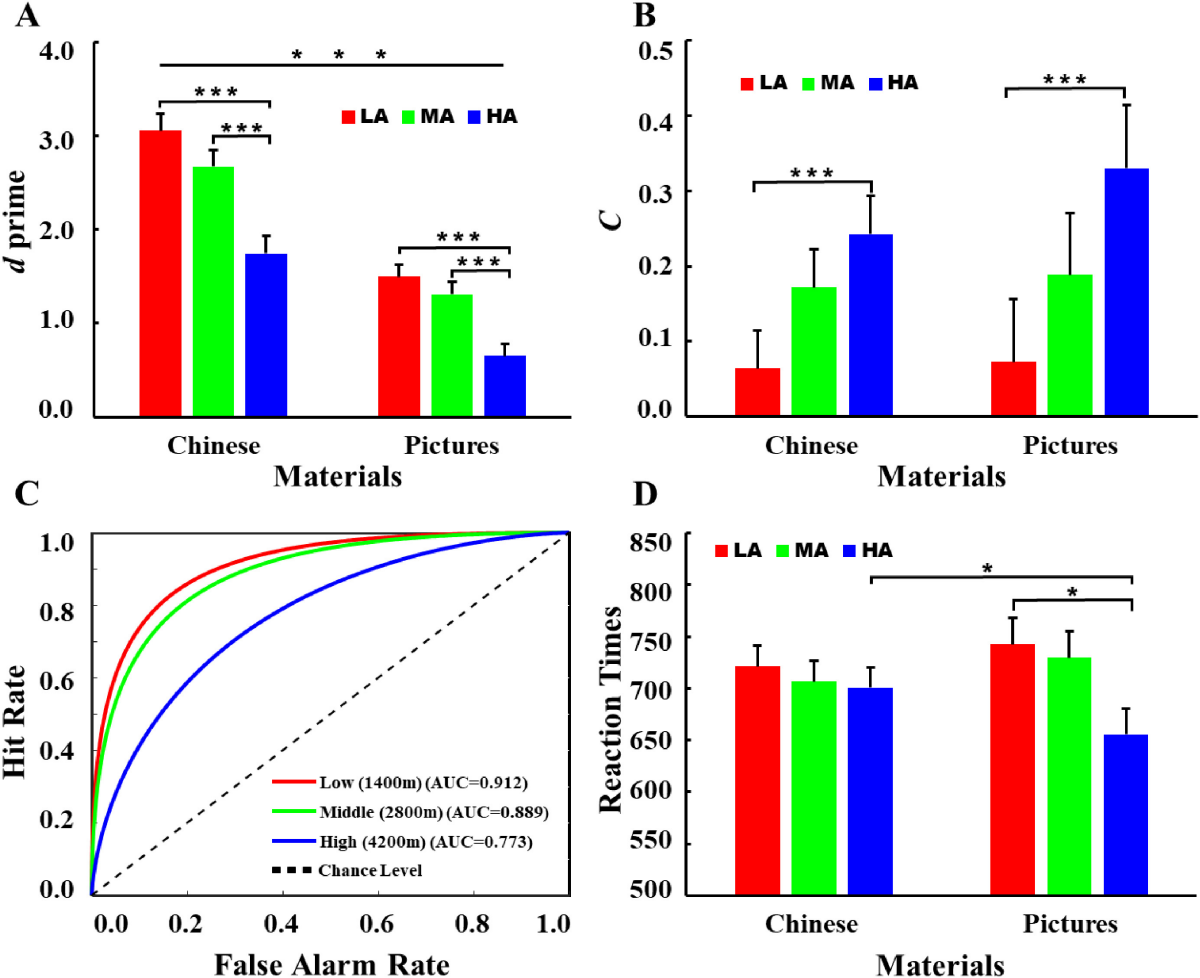
Behavioral data of participants across three altitude groups. **(A)** *d’*; **(B)** *C* index; **(C)** receiver operating characteristic (ROC) curves, and **(D)** reaction times. ROC curves for the three altitude groups based on signal detection performance. ROC curves were constructed using individual hit rates (HR) and false alarm rates (FAR) calculated after excluding trials with no response. The area under the curve (AUC) was computed using a non-parametric trapezoidal integration method. In addition, for the analysis of RTs, only trials with correct behavioral responses were included. This encompassed both “hit” trials (i.e., correctly identifying an old stimulus by pressing the “F” key) and “correct rejection” trials (i.e., correctly identifying a new stimulus by pressing the “J” key). LA, Low Altitude (1,400 m); MA, Mid Altitude (2,800 m); HA, High Altitude (4,200 m). Error bars represent ± SEM. *p < 0.05, ***p < 0.001.

Descriptive statistics for hit rates and false alarm rates are presented in Table 2. A 3 (Altitude: LA, MA, HA) × 2 (Material type: Picture, Chinese Character) repeated measures ANOVA was conducted on the *d’* and *C* indices (Figure 3). For *d’*, there was a significant main effect of altitude, *F*(2, 74) = 21.71, *p* < 0.001, η*_*p*_^2^* = 0.37. The high-altitude group exhibited significantly lower *d’* values (1.20 ± 0.12) than both the low-altitude group [2.27 ± 0.12, *p* < 0.001, 95% CI = (-1.49, -0.66)] and the mid-altitude group [2.00 ± 0.12, *p* < 0.001, 95% CI = (-1.21, -0.38)]. No significant difference was found between the low- and mid-altitude groups [2.27 ± 0.12 vs. 2.00 ± 0.12, *p* = 0.29, 95% CI = (-0.13, 0.69)]. The main effect of material type was significant, *F*(1, 74) = 129.45, *p* < 0.001, η*_*p*_^2^* = 0.64. Chinese character materials exhibited significantly higher *d’* values (2.49 ± 0.10) than picture materials [1.15 ± 0.08, *p* < 0.001, 95% CI = (1.10, 1.57)]. No significant interaction between altitude and material type was found, *F*(2, 74) = 1.30, *p* = 0.28, η*_*p*_^2^* = 0.034. For the *C* index, a significant main effect of altitude was found, *F*(2, 74) = 4.40, *p* = 0.016, η*_*p*_*^2^ = 0.11. The high-altitude group had a significantly higher *C* value (0.29 ± 0.05) than the low-altitude group [0.07 ± 0.05, *p* = 0.012, 95% CI = (0.04, 0.40)], with no difference between low- and mid-altitude groups [0.07 ± 0.05 vs. 0.18 ± 0.05, *p* = 0.39, 95% CI = (-0.29, 0.07)], as well as between high- and mid-altitude groups [0.29 ± 0.05 vs. 0.18 ± 0.05, *p* = 0.46, 95% CI = (-0.07, 0.29)] were found. Neither the main effect of material [*F*(1, 74) = 0.53, *p* = 0.47, η*_*p*_*^2^ = 0.007] nor the altitude × material interaction [*F* (2, 74) = 0.23, *p* = 0.80, η*_*p*_*^2^ = 0.006] was significant.

**TABLE 2:** Descriptive statistics for hit rates and false alarm rates.

Material type	Altitude (m)	HR (mean ± SD)	FAR (mean ± SD)	N
Chinese characters	1,400	0.92 ± 0.08	0.05 ± 0.07	26
	2,800	0.85 ± 0.15	0.08 ± 0.10	26
	4,200	0.71 ± 0.22	0.15 ± 0.16	25
Pictures	1,400	0.73 ± 0.19	0.22 ± 0.12	26
	2,800	0.67 ± 0.18	0.23 ± 0.18	26
	4,200	0.45 ± 0.25	0.26 ± 0.18	25

HR, hit rate; FAR, false alarm rate. Values are presented as mean ± standard deviation. Only trials with valid responses were included in the calculation.

Based on the group-averaged *d*′ and *C*, we further characterized the theoretical receiver operating characteristic (ROC) curves for the three altitude groups and calculated the area under the curve (AUC) using the trapezoidal integration method (Trapz function) to examine between-group differences in discriminative ability. The theoretical ROC curves were plotted using the function HR = Φ(*d’* + Φ - FAR), where HR denotes the hit rate and FAR denotes the false alarm rate. A curve closer to the upper-left corner of the ROC space indicates stronger discriminative ability, and a larger AUC reflects better overall discriminative performance. As shown in Figure 3C, the AUC values for the low-, mid-, and high-altitude groups were 0.91, 0.89, and 0.77, respectively.

A 3 (Altitude: LA, MA, HA) × 2 (Material: Picture, Chinese Character) repeated measures ANOVA conducted on RTs revealed no significant main effects of either altitude [*F*(2, 74) = 1.82, *p* = 0.17, η*_*p*_*^2^ = 0.05] or material [*F*(1, 74) = 0.001, *p* = 0.97, η*_*p*_*^2^ = 0.000]. A significant altitude × material interaction was found, *F*(2, 74) = 4.53, *p* = 0.014, η*_*p*_*^2^ = 0.11. Simple effects analysis revealed that in the high-altitude group, RTs for Chinese character materials (700.34 ± 19.91 ms) were significantly longer than those for picture materials [655.23 ± 25.64 ms, *p* = 0.016, 95% CI = (8.58, 81.65)]. No significant differences in RTs between the two materials were observed in the low- and mid-altitude groups. To further decompose this interaction, we examined the simple effects of altitude for each material type. For Chinese character materials, no significant differences in RTs were found among the three altitude groups. In contrast, for picture materials, a significant effect of altitude was observed: the low-altitude group (742.58 ± 25.15 ms) exhibited significantly longer RTs than the high-altitude group [655.23 ± 25.64 ms *p* = 0.052, 95% CI = (-0.61, 175.31)], whereas no significant differences were detected between the mid-altitude group and either the low- or high-altitude groups. Note that for the analysis of RTs, only trials with correct behavioral responses were included. This encompassed both “hit” trials (i.e., correctly identifying an old stimulus by pressing the “F” key) and “correct rejection” trials (i.e., correctly identifying a new stimulus by pressing the “J” key).

### 3.4 ERP results

It is noteworthy that ERP averaging was performed exclusively on trials with correct behavioral responses following Zhang et al. (2019), and the retrieval-stage analysis combined “new” and “old” stimuli without examining the old/new effect. This approach was adopted because further subdivision into correct/incorrect and old/new conditions would have resulted in fewer than 20 valid trials per condition, thereby compromising the reliability and signal-to-noise ratio of ERP averages.

#### 3.4.1 Encoding stage

**FIGURE 4:**
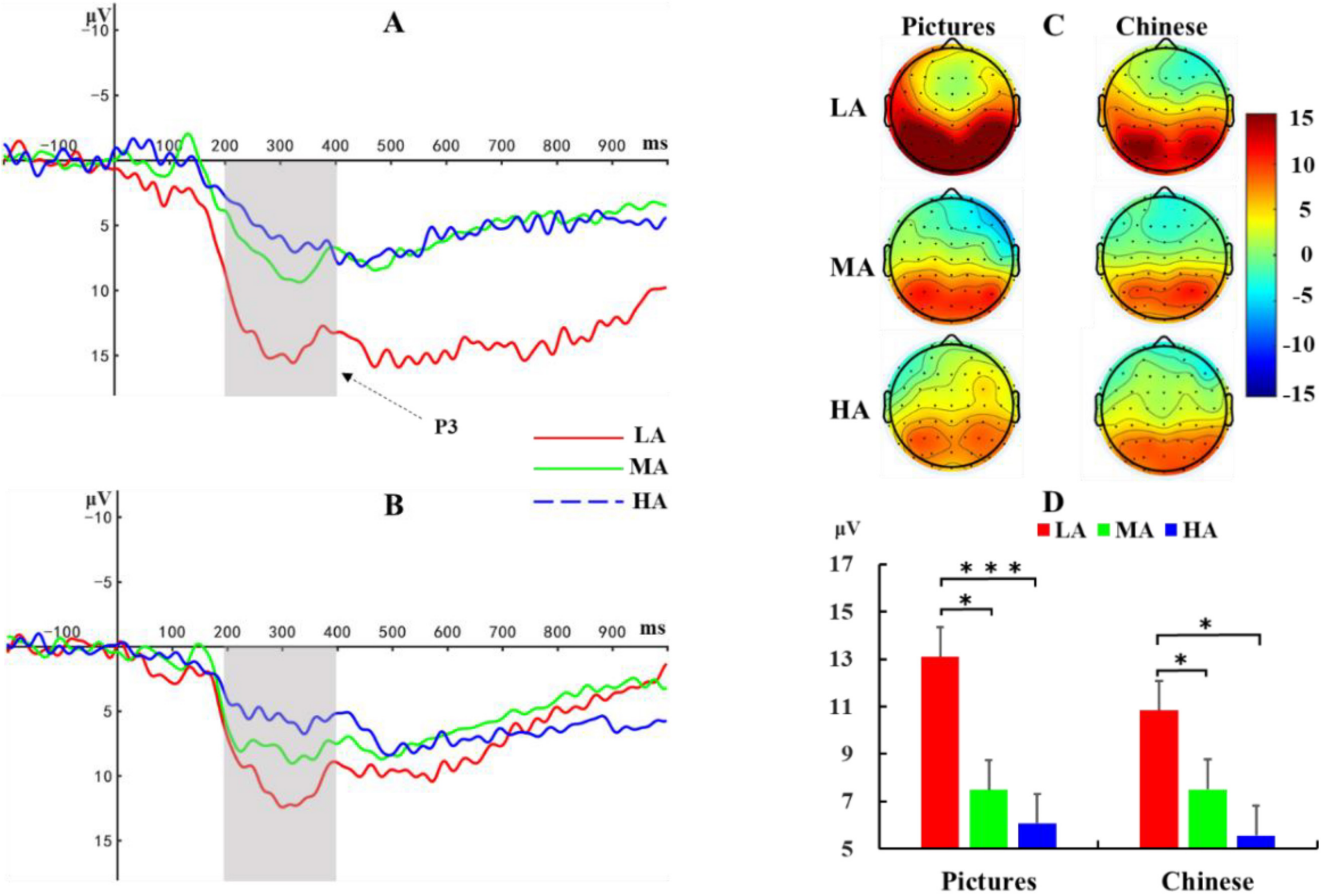
ERP waveforms during the memory encoding stage. **(A)** ERP responses to picture stimuli. **(B)** ERP responses to Chinese character stimuli. **(C)** Topographic maps of P3 amplitudes averaged across six electrode sites (P1, Pz, P2, CP1, CPz, CP2) within the 200–400 ms time window for both stimulus types across the three altitude groups. **(D)** Statistical comparison of P3 amplitudes. LA, Low Altitude (1,400 m); MA, Mid Altitude (2,800 m); HA, High Altitude (4,200 m). Error bars indicate standard error. *p < 0.05, ***p < 0.001.

Based on previous research (Zhang et al., 2019) and visual inspection of waveform plots, the P3 component in this study was defined as the average amplitude across six electrode sites (P1, Pz, P2, CP1, CPz, CP2) within the 200–400 ms time window. A 3 (Altitude: LA, MA, HA) × 2 (Material type: Picture, Chinese Character) repeated measures ANOVA was conducted on P3 amplitude. Results (Figure 4) showed a significant main effect of altitude, *F*(2, 72) = 9.65, *p* < 0.001, η*_*p*_*^2^ = 0.21. *Post hoc* tests revealed that the low-altitude group exhibited significantly higher P3 amplitude (11.97 ± 1.02 μV) than both the mid-altitude group [7.50 ± 1.02 μV, *p* = 0.003, 95% CI = (1.58, 7.35)] and the high-altitude group [5.82 ± 1.02 μV, *p* < 0.001, 95% CI = (3.26, 9.03)]. No significant difference was observed between the mid- and high-altitude groups in P3 amplitude. The main effect of material was not significant, *F*(1, 72) = 1.28, *p* = 0.26, η*_*p*_*^2^ = 0.02, nor was the altitude × material interaction, *F*(2, 72) = 0.71, *p* = 0.50, η*_*p*_*^2^ = 0.02.

#### 3.4.2 Maintenance stage

**FIGURE 5:**
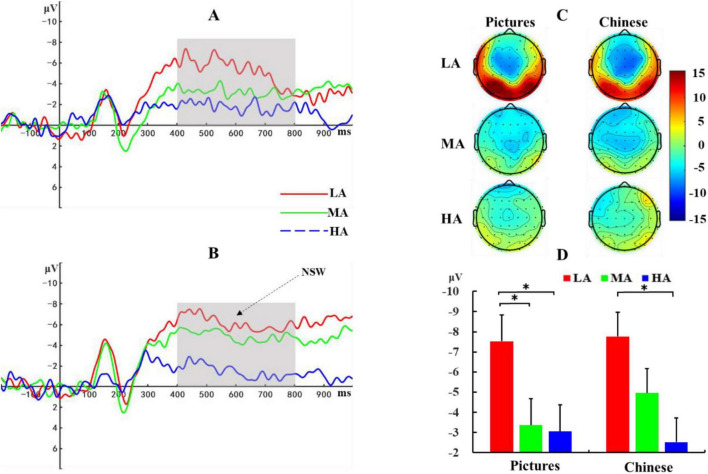
ERP waveforms during the memory maintenance stage. **(A)** ERP responses to picture stimuli. **(B)** ERP responses to Chinese character stimuli. **(C)** Topographic maps of negative slow wave (NSW) amplitudes averaged across six electrode sites (F1, Fz, F2, FC1, FCz, FC2) within the 400–800 ms time window for both stimulus types across the three altitude groups. **(D)** Statistical comparison of NSW amplitudes. LA, Low Altitude (1,400 m); MA, Mid Altitude (2,800 m); HA, High Altitude (4,200 m). Error bars indicate standard error. *p < 0.05.

Based on previous research (Zhang et al., 2019) and visual inspection of waveform plots, the NSW component in this study was defined as the average amplitude across six electrode sites (F1, Fz, F2, FC1, FCz, FC2) within the 400–800 ms time window. A 3 (Altitude: LA, MA, HA) × 2 (Material: Picture, Chinese Character) repeated measures ANOVA was conducted on NSW amplitude. Results (Figure 5) showed a significant main effect of altitude, *F*(2, 72) = 7.01, *p* = 0.002, η*_*p*_^2^* = 0.16. *Post-hoc* comparisons revealed that the low-altitude group exhibited a more negative NSW amplitude (-7.65 ± 0.95 μV) compared to both the mid-altitude group [-4.16 ± 0.95 μV, *p* = 0.01, 95% CI = (-6.16, -0.82)] and the high-altitude group [-2.79 ± 0.95 μV, *p* < 0.001, 95% CI = (-7.53, -2.20)]. No significant difference in NSW amplitude was observed between the mid- and high-altitude groups. The main effect of material was not significant, *F*(1, 72) = 0.20, *p* = 0.65, η*_*p*_*^2^ = 0.003, nor was the altitude × material interaction, *F*(2, 72) = 0.42, *p* = 0.66, η*_*p*_*^2^ = 0.01.

#### 3.4.3 Retrieval stage

**FIGURE 6:**
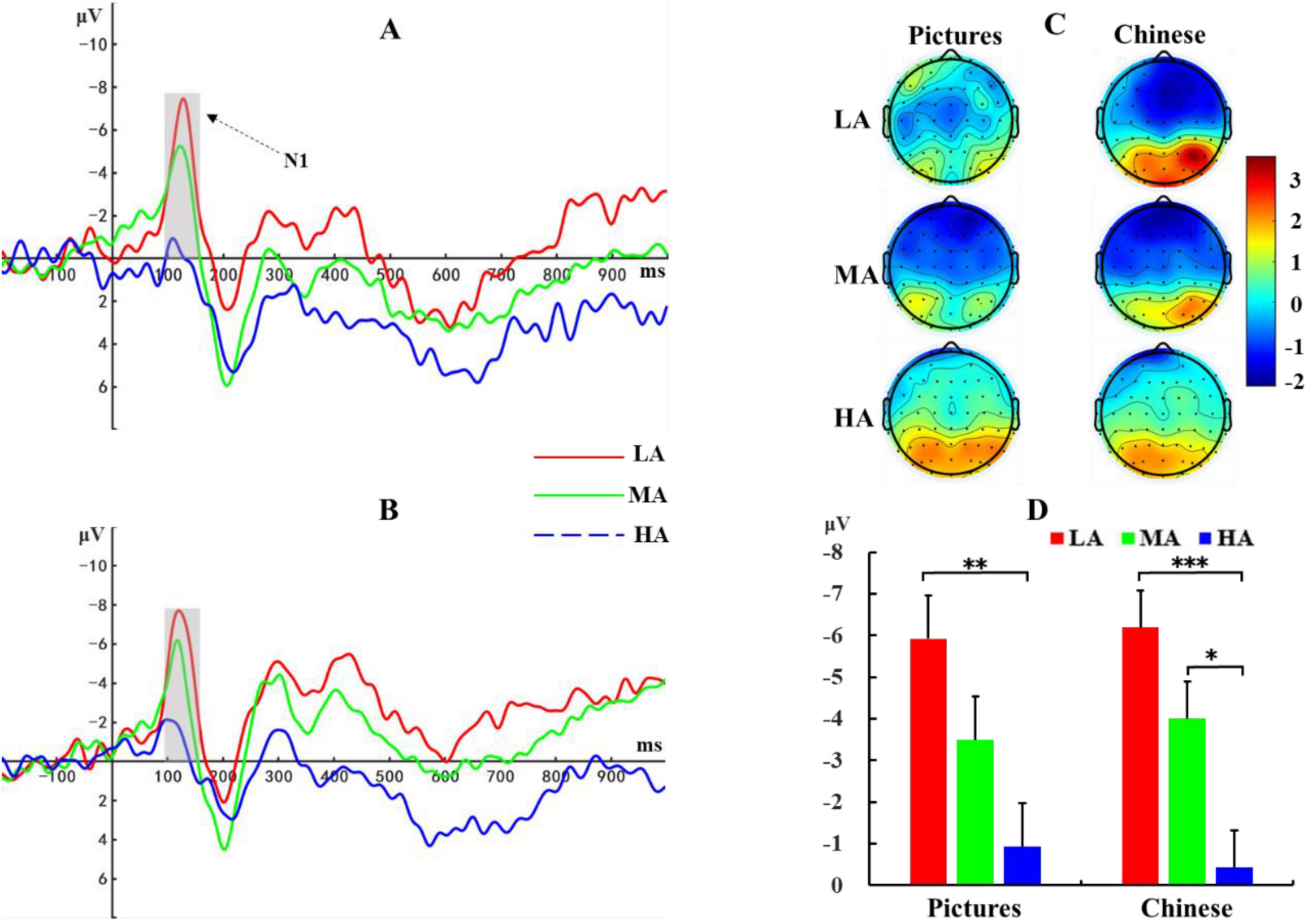
ERP waveforms during the memory retrieval stage. **(A)** ERP responses to picture stimuli. **(B)** ERP responses to Chinese character stimuli. **(C)** Topographic maps of N1 amplitudes averaged across three electrode sites (Fz, FCz, Cz) within the 100–160 ms time window for both stimulus types across the three altitude groups. (D) Statistical comparison of N1 amplitudes. LA, Low Altitude (1,400 m); MA, Mid Altitude (2,800 m); HA, High Altitude (4,200 m). Error bars indicate standard error. *p < 0.05, **p < 0.01, ***p < 0.001.

Based on previous research (Barkaszi et al., 2016) and visual inspection of waveform plots, the N1 component in this study was defined as the average amplitude across three electrode sites (Fz, FCz, Cz) within the 100–160 ms time window. A 3 (Altitude: LA, MA, HA) × 2 (Material: Picture, Chinese Character) repeated measures ANOVA was conducted on N1 amplitude. Results (Figure 6) showed a significant main effect of altitude, *F* (2, 72) = 9.88, *p* < 0.001, η*_*p*_*^2^ = 0.22. *Post hoc* tests revealed that both the low-altitude group [-6.06 ± 0.86 μV, *p* < 0.001, 95% CI = (-7.80, -2.96)] and mid-altitude group [-3.75 ± 0.86 μV, *p* = 0.01, 95% CI = (-5.50, -0.65)] exhibited more negative N1 amplitudes compared to the high-altitude group (-0.67 ± 0.86 μV). No significant difference in N1 amplitude was observed between the low- and mid-altitude groups. The main effect of material was not significant, *F*(1, 72) = 0.04, *p* = 0.85, η*_*p*_*^2^ < 0.001, nor was the altitude × material interaction, *F*(2, 72) = 0.35, *p* = 0.70, η*_*p*_*^2^ = 0.01.

### 3.5 Correlations between behavioral/EEG indices and physiological indices

To further investigate the relationship between chronic high-altitude hypoxia and memory function, we conducted correlation analyses among physiological indices, behavioral indices, and EEG indices. Given the large number of correlation analyses, false discovery rate (FDR) correction (Benjamini–Hochberg procedure) was applied to control for multiple comparisons. After correction, the adjusted significance threshold was set at *p* < 0.016. Only correlations surviving FDR correction were considered statistically significant.

After FDR correction, several significant correlations were observed among physiological, behavioral, and electrophysiological indices (Table 3). SpO_2_ showed significant positive correlations with the discriminability index (d’) for both Chinese character and picture materials, indicating that higher oxygen saturation was associated with better recognition sensitivity. At the electrophysiological level, SpO_2_ was significantly and positively correlated with P3 amplitude during encoding for both stimulus types, and significantly negatively correlated with NSW amplitude during maintenance and N1 amplitude during retrieval. These findings suggest that reduced oxygen availability is associated with altered neural activity across multiple stages of memory processing. Heart rate was significantly and positively correlated with P3 amplitude for picture stimuli only, whereas MVC did not show any significant correlations after FDR correction. In addition, significant correlations were observed among behavioral indices, including negative associations between decision criterion (*C*) and *d’*, as well as a positive correlation between *d’* values for picture and Chinese character materials, indicating consistency in individual recognition sensitivity across stimulus types.

**TABLE 3:** Correlations between behavioral/EEG indices and physiological measures.

	SpO_2_	MVC	HR	*C*_C	*C*_P	*d*′_C	*d*′_P
*C*_C	–0.27 (0.010)*	–0.05 (0.65)	–0.11 (0.31)	1	1	1	1
*C*_P	–0.21 (0.05)	–0.14 (0.20)	–0.05 (0.65)	–0.35 (0.001)*			
*d*′_C	0.34 (0.001)*	0.18 (0.09)	0.21 (0.05)	–0.25 (0.02)	–0.35 (0.001)*		
*d*′_P	0.31 (0.003)*	0.23 (0.03)	0.05 (0.66)	–0.07 (0.49)	–0.32 (0.003)*	0.43 (0.000)*	
P3_C	0.30 (0.010) *	0.19 (0.11)	0.12 (0.32)	–0.21 (0.08)	–0.04 (0.71)	0.01 (0.91)	0.14 (0.22)
P3_P	0.35 (0.002)*	0.08 (0.49)	0.36 (0.002)*	–0.19 (0.11)	0.09 (0.45)	0.09 (0.45)	0.02 (0.89)
NSW_C	–0.34 (0.003)*	–0.10 (0.42)	–0.24 (0.04)	–0.01 (0.94)	–0.04 (0.73)	0.05 (0.67)	0.06 (0.61)
NSW_P	–0.33 (0.004)*	0.13 (0.27)	–0.06 (0.62)	–0.16 (0.17)	–0.06 (0.61)	0.02 (0.89)	0.05 (0.69)
N1_C	–0.36 (0.002)*	–0.05 (0.69)	–0.18 (0.21)	0.09 (0.51)	0.16 (0.18)	–0.05 (0.69)	–0.20 (0.11)
N1_P	–0.28 (0.016)*	0.09 (0.43)	0.02 (0.85)	0.06 (0.62)	0.04 (0.73)	–0.06 (0.63)	–0.05 (0.67)

Pearson correlation coefficients are shown, with *p-*values in parentheses. Significant correlations after false discovery rate (FDR) correction (Benjamini–Hochberg procedure) are marked with *. The corrected significance threshold was *p* < 0.016. SpO_2_, blood oxygen saturation; MVC, maximal vital capacity; HR, heart rate; *C*_C/*C*_P, response criterion (C) for Chinese character materials/picture; *d’*_C/*d’*_P, discriminability index (*d’*) for Chinese character materials/picture; P3_C/P3_P, P3 amplitude during encoding stage for Chinese character materials/picture; NSW, negative slow wave; NSW_P/NSW_C, NSW amplitude during maintenance stage for Chinese character materials/picture; N1_P/N1_C, N1 amplitude during retrieval stage for Chinese character materials/picture.

## 4 Discussion

By establishing three altitude groups—low- (1,400 m), mid- (2,800 m), and high-altitude (4,200 m)—and integrating physiological, behavioral, and EEG measures, this study investigated the effects of chronic high-altitude exposure on memory function and neural processing in indigenous highland junior high school students using a delayed recognition task.

### 4.1 Physiological indices

Previous studies have shown that individuals newly exposed to high altitudes typically exhibit a range of acute compensatory responses to hypoxia, including increased pulmonary ventilation, elevated heart rate and cardiac output, heightened blood pressure, and increased red blood cell and hemoglobin concentrations (Chen and He, 2025; Sheng et al., 2021; Wang et al., 2023). These responses are widely interpreted as short-term mechanisms aimed at maintaining oxygen delivery under sudden hypoxic stress. However, comparatively little research has examined how chronic high-altitude exposure affects physiological regulation in long-term native residents. In the present study, SpO_2_ declined systematically with increasing altitude, reflecting progressively reduced environmental oxygen availability. In contrast, heart rate showed only a marginal altitude-related effect and exhibited a decreasing trend. Although the low-altitude group displayed a higher resting heart rate than the high-altitude group, the overall effect did not reach conventional levels of statistical significance. This pattern differs from the elevated heart rate commonly observed during acute hypoxic exposure and suggests the presence of long-term adaptive regulation (Hogan et al., 2010; Richardson et al., 2011). Importantly, the tendency toward lower resting heart rate at higher altitudes may reflect a shift from short-term “stress compensation” to long-term “energy-saving optimization” in indigenous populations. Unlike acute exposure, which is characterized by sympathetic activation and tachycardia, long-term residents appear to develop more efficient cardiovascular regulation that supports adequate oxygen delivery at lower energetic cost. This interpretation is consistent with evidence showing enhanced stroke volume, improved myocardial efficiency, and increased parasympathetic dominance in high-altitude natives (Kamat and Banerji, 1972; Wang et al., 2025). In addition, elevated nitric oxide bioavailability in high-altitude populations promotes systemic vasodilation and reduces cardiac afterload, thereby enabling effective circulation without excessive increases in heart rate (Erzurum et al., 2007; Hoit et al., 2005).

Notably, MVC also decreased with increasing altitude in our native adolescent sample. At first glance, this finding appears inconsistent with studies reporting increased ventilatory capacity and elevated resting heart rate in lowland migrants during short-term or subacute acclimatization. However, accumulating evidence indicates that these increases primarily reflect transient compensatory responses rather than stable adaptations. In contrast, long-term high-altitude natives exhibit distinct physiological phenotypes shaped by prolonged hypoxic exposure. Specifically, high-altitude natives tend to develop enhanced cardiac stroke volume and more efficient myocardial function, allowing adequate tissue oxygen delivery at lower resting heart rates (Wang et al., 2025). In parallel, elevated hemoglobin concentration and optimized oxygen transport capacity improve systemic oxygen utilization (Kamat et al., 1972). At the respiratory level, long-term adaptation is associated with improved ventilatory efficiency and reduced reliance on high ventilation volumes, which may explain the observed reduction in MVC (Song et al., 2025). Moreover, metabolic and genetic studies suggest that high-altitude populations exhibit reduced basal metabolic rate and enhanced oxygen utilization efficiency, partly mediated by hypoxia-related genes such as EPAS1 (Simonson et al., 2010). These adaptations further support an energy-conserving physiological strategy under chronic hypoxic conditions.

Together, these cardiovascular and respiratory adaptations contribute to an energy-efficient and sustainable physiological equilibrium characterized by relatively low resting heart rate and moderate ventilatory demand. Rather than reflecting functional decline, this pattern represents a stable “low-consumption” homeostatic state optimized for chronic hypoxic environments. This adaptive profile fundamentally distinguishes long-term native residents from recently acclimatized migrants, who primarily rely on energetically costly short-term responses such as hyperventilation and tachycardia.

Therefore, the reduced heart rate and MVC observed in the present study are best interpreted as indicators of long-term physiological optimization rather than impairment. These findings are consistent with classical models of high-altitude adaptation and underscore the importance of distinguishing between acute acclimatization and chronic native adaptation when interpreting altitude-related physiological changes. Nevertheless, it should be noted that the present study did not directly assess autonomic regulation (Zhuang et al., 1993), nitric oxide metabolism, or genetic markers related to hypoxia adaptation. As a result, the proposed mechanistic interpretations remain inferential and should be validated in future investigations using more comprehensive physiological and molecular assessments.

### 4.2 Behavioral results

Descriptive statistics for hit rates (HR) and false alarm rates (FAR) are presented in Table 2. Across both Chinese character and picture materials, HR showed a decreasing trend with increasing altitude, whereas FAR exhibited a corresponding increase. This pattern indicates a progressive reduction in correct recognition accompanied by elevated false alarms under higher-altitude conditions. These changes jointly contributed to the observed decline in discriminability, as reflected by reduced *d’* values. The analysis of behavioral performance, incorporating both the *d’* and RTs, revealed a distinct pattern among participants at high altitude. Specifically, while individuals in the high-altitude group generally demonstrated lower *d’* values relative to their low- and mid-altitude counterpartslly demonstrated lower ants at high altitude. Specifically, while individuals in the high accompanied by elevated false alarms undepared to Chinese characters. This was reflected in both a marginally higher *d’* and significantly shorter RTs for pictures versus characters within the high-altitude group. This pattern suggests that under high-altitude conditions, individuals may rely more efficiently on perceptual processing associated with pictorial materials, as opposed to the more semantically demanding processing required for Chinese characters. Since *d’* reflects an individual’s sensitivity in distinguishing target signals (old items) from noise (new items) (Tanner and Swets, 1954; Wixted, 2020), the general depression in *d’* at high altitude points to an impairment in overall recognition accuracy. However, the within-group advantage for pictures implies that perceptually based stimuli are comparatively less affected by such impairment. This interpretation aligns with existing evidence indicating that Chinese characters, as meaningful verbal stimuli, engage deeper semantic and cognitive processing compared to pictures, which are often processed as holistic, non-verbal units (Xiao, 2008). The observed RT advantage for pictures further supports the notion of more efficient encoding and retrieval for perceptual materials under cognitively demanding conditions such as high-altitude hypoxia. Thus, although chronic hypoxia may degrade overall discriminability, it appears to shift processing preference toward less resource-intensive, perceptually driven stimuli.

For the decision criterion index (*C*), a similar pattern as *d’* emerged: The *C* values for the low- and mid-altitude groups were significantly lower than those of the high-altitude group, again with no significant difference between the low- and mid-altitude groups. According to signal detection theory, *C* represents an individual’s response bias or decision threshold. Higher *C* values reflect a more conservative decision-making strategy—requiring stronger evidence before categorizing a stimulus as previously seen—whereas lower *C* values indicate a more liberal response bias (Tanner and Swets, 1954; Wixted, 2020). The current results therefore suggest that high-altitude participants adopted a more conservative response strategy, characterized by lower hit rates and higher miss rates, compared to participants in the lower-altitude groups.

By constructing theoretical ROC curves (Figure 3C) and calculating the AUC based on group-averaged *d’* and *C* values, a clear altitude-dependent gradient emerged. Specifically, AUC values decreased progressively with increasing altitude. The ROC curves for the low- and mid-altitude groups were situated closer to the upper-left corner of the plot, indicating superior overall discriminative performance relative to the high-altitude group. Taken together, the indices of *d’*, *C*, and ROC analysis collectively suggest that participants in the high-altitude group exhibited weaker overall signal discrimination ability and a more conservative decision-making bias compared to those in the low- and mid-altitude groups.

### 4.3 Discussion of EEG results

During the encoding stage, this study found that the P3 amplitude in the low-altitude group was significantly higher than that in the mid- and high-altitude groups, with no significant difference between the mid- and high-altitude groups. The P3 component is typically associated with the allocation of cognitive resources and reflects elaborate evaluation of stimuli (Ito et al., 1998; Shang, 2013). The larger P3 amplitude in the low-altitude group may indicate that these participants allocated more cognitive resources during memory encoding, leading to deeper stimulus processing. In contrast, the smaller P3 amplitudes in the mid- and high-altitude groups may reflect insufficient cognitive resource allocation or reduced attention concentration. Additionally, P3 amplitude is closely linked to the success of memory encoding: successfully encoded stimuli generally evoke larger P3 amplitudes, while failed encoding results in smaller P3 amplitudes (Rugg et al., 1998). The larger P3 amplitude in the low-altitude group suggests higher encoding efficiency during memory encoding, enabling more effective transformation of stimulus information into memory traces. This indicates stronger cognitive resource allocation ability and higher memory encoding efficiency in the low-altitude group. The smaller P3 amplitudes in the mid- and high-altitude groups may reflect reduced memory encoding efficiency, possibly due to the negative impact of chronic high-altitude exposure on the memory encoding process.

Previous studies have shown that the NSW is an ERP component closely associated with memory maintenance (Khader et al., 2007; Ruchkin et al., 2003; Vogel and Machizawa, 2004), typically appearing about 400 ms after stimulus presentation as a negative slow wave that persists throughout the memory maintenance stage (Wang et al., 2016). A previous research indicated that the amplitude of NSW correlates with the strength of memory maintenance, where a more negative amplitude generally signifies better memory maintenance (Chen et al., 2018). In this study, during the maintenance stage, the low-altitude group exhibited a more negative NSW amplitude than the mid- and high-altitude groups, with no significant difference between the mid- and high-altitude groups. This suggests that the low-altitude group had stronger memory maintenance ability during the maintenance stage compared to the mid- and high-altitude groups. These findings align with previous research; for example, a previous study has found that long-term high-altitude exposure only negatively impacted response inhibition and information maintenance abilities among natives residing at 4,200 m, indicating that an altitude of approximately 4,000 m may be a threshold for spatial working memory impairment in Tibetan natives (Ma et al., 2020).

Finally, during the memory retrieval stage, statistical analysis of the N1 component revealed that within the 100–160 ms time window after retrieval task presentation, the low- and mid-altitude groups evoked more negative N1 amplitudes than the high-altitude group. As an early ERP component, N1 typically appears 80–150 ms after stimulus presentation and reflects the brain’s early perception and selective attention processes for stimuli (Ward and Beaman, 2024). In memory retrieval tasks, N1 may reflect the early recognition process of target stimuli (Luck, 2005; Vogel and Luck, 2000). The more negative N1 amplitudes in the low- and mid-altitude groups compared to the high-altitude group suggest that the high-altitude group had reduced early perception and attention capture abilities.

An integrated examination of EEG indices across the encoding, maintenance, and retrieval stages of memory revealed that the effects of chronic hypoxic exposure on memory function were jointly modulated by altitude and memory stage. Specifically, in the mid-altitude group, chronic hypoxia significantly impaired memory encoding (i.e., the allocation of attentional resources) and maintenance (i.e., the capacity for information maintenance), but had no significant effect on the retrieval stage (i.e., early perceptual processing and attentional capture). This stage-specific impairment in the mid-altitude group was also reflected behaviorally, as no significant differences in memory performance were observed between the low- and mid-altitude groups across behavioral indices such as *d’*, *C*, and ROC curves. In contrast, the high-altitude group exhibited impairments across all three stages, suggesting that chronic hypoxic exposure at high altitudes adversely affected attentional allocation during encoding, information maintenance during the delay period, and early perceptual and attentional responses during retrieval. Overall, compared to their low- and mid-altitude counterparts, high-altitude participants allocated fewer attentional resources to target stimuli during encoding, exhibited reduced capacity to maintain information during the delay period, and demonstrated diminished early perceptual and attentional responses during retrieval.

### 4.4 Discussion of correlation analysis results

To clarify the relationships among physiological status, behavioral performance, and neural activity, correlation analyses were conducted with FDR correction. After correction, only a limited number of associations remained statistically significant, primarily involving SpO_2_, behavioral discriminability (*d’*), and selected ERP components (Table 3). This pattern indicates that although physiological, behavioral, and electrophysiological measures are interrelated, most associations were modest in magnitude and should be interpreted cautiously. Specifically, SpO_2_ showed significant positive correlations with d’ for both Chinese character and picture materials, suggesting that higher oxygen saturation was associated with better recognition sensitivity. In addition, SpO_2_ was positively correlated with P3 amplitude during encoding and negatively correlated with NSW amplitude during maintenance and N1 amplitude during retrieval for both stimulus types. In contrast, most correlations involving heart rate and MVC did not survive FDR correction, with the exception of a positive association between heart rate and P3 amplitude for picture stimuli.

These findings suggest that reduced oxygen availability under chronic high-altitude exposure may compromise neural efficiency across multiple memory stages. Lower SpO_2_ likely limits metabolic support for neural signaling, thereby reducing attentional resource allocation during encoding, disrupting sustained activity during maintenance, and constraining early perceptual processing during retrieval. This interpretation is consistent with the observed associations between SpO_2_, P3, NSW, and N1 amplitudes. Taken together, the correlation patterns provide preliminary evidence that chronic hypoxia influences memory performance through its impact on neural processing efficiency. However, the limited number of significant associations after correction indicates that these effects are not uniformly strong and may vary across individuals and task components.

Furthermore, considering that the three altitude groups were located at approximately 1,400, 2,800, and 4,200 m, the convergence of behavioral impairments, ERP alterations, and SpO_2_-related correlations in the highest-altitude group suggests that more pronounced cognitive changes may emerge above 4,000 m. This pattern is broadly consistent with previous reports of neurocognitive alterations in plateau residents living at similar elevations (Berry et al., 1989; Evans and Witt, 1966; Ma et al., 2017, 2020). Nevertheless, because only three discrete altitude levels were examined, the proposed “critical altitude” should be regarded as preliminary. From a physiological and cognitive perspective, chronic reductions in oxygen saturation may not be fully compensated by long-term cardiovascular and respiratory adaptations, particularly during cognitively demanding tasks. Incomplete compensation may reduce oxygen delivery to frontal and parietal networks supporting attention and working memory, thereby contributing to impairments in encoding, maintenance, and retrieval. Overall, these results indicate that chronic hypoxia affects adolescent memory function through both direct physiological constraints and indirect effects on neural efficiency. Although partial adaptation may occur in long-term residents, it appears insufficient to fully preserve cognitive performance under higher hypoxic loads.

## 5 Conclusion, limitations, and future directions

Using indigenous junior high school students residing on the Qinghai–Tibet Plateau as participants, the present study systematically examined the effects of chronic hypoxic exposure at different altitudes on memory function and neural processing using a delayed recognition paradigm. By integrating physiological, behavioral, and electrophysiological indices, the following conclusions can be drawn. First, with increasing altitude, participants’ SpO_2_ and MVC showed significant decreases, while heart rate exhibited a declining trend. These findings suggest progressive physiological adaptation to chronic hypoxic environments. Second, chronic exposure to high-altitude hypoxia was associated with impaired memory performance in adolescent residents, as reflected in reduced behavioral discriminability and altered ERP components across encoding, maintenance, and retrieval stages. This indicates that the adverse effects of hypoxia on memory are stage-specific and involve multiple neural processes. Third, participants residing at 4,200 m exhibited consistently poorer behavioral and electrophysiological performance than those living at lower altitudes. When considered alongside previous findings, this pattern provides preliminary support for the notion that cognitive vulnerability may increase markedly at altitudes around 4,000 m. However, this inference should be interpreted with caution given the limited number of altitude levels examined.

Despite these findings, several limitations should be acknowledged. First, the present study employed a cross-sectional and correlational design, which precludes firm causal inferences regarding the effects of chronic hypoxia on cognition. Longitudinal and experimental studies are therefore needed. Second, participants were recruited using convenience sampling from a limited number of schools for logistical reasons related to EEG data collection. Although age, grade level, and general intelligence were controlled, other factors such as socioeconomic background and educational environment could not be fully accounted for, introducing potential selection bias. Third, the sample size was relatively modest, and no *a priori* power analysis was conducted. Data collection was constrained by regional educational schedules, including the annual “Cordyceps holiday,” which may have limited statistical power and generalizability. Fourth, anthropometric variables (e.g., height, weight, and body mass index) were not systematically assessed, despite their potential influence on physiological and cognitive outcomes. Fifth, data were collected at different time points across altitude groups due to fieldwork logistics, and seasonal or contextual effects cannot be entirely excluded. Finally, direct measures of autonomic regulation, nitric oxide metabolism, basal metabolic rate, and hypoxia-related genetic markers were not included. As a result, the physiological mechanisms underlying altitude-related changes in heart rate and oxygen utilization could only be inferred indirectly, limiting the strength of mechanistic interpretations.

Future research should address these limitations by recruiting larger and more diverse samples, adopting longitudinal designs, and incorporating comprehensive physiological and anthropometric assessments. In particular, future studies should integrate measures of autonomic regulation, vascular function, metabolic efficiency, and genetic variation to directly examine the biological mechanisms underlying long-term hypoxia adaptation. Including multiple age groups and finer altitude gradients, combined with repeated follow-up assessments, would help clarify developmental trajectories and potential threshold effects of chronic hypoxia and distinguish environmental from maturational influences. Moreover, the integration of multimodal neuroimaging with high temporal and spatial resolution, together with heart rate variability, nitric oxide–related biomarkers, and genomic profiling, may provide a more comprehensive framework for understanding the interactions between physiological adaptation and cognitive development. Such efforts will contribute to a more systematic and mechanistically grounded understanding of how long-term high-altitude exposure influences brain function and cognition in adolescent populations.

## Data Availability

The original contributions presented in the study are included in the article/supplementary material, further inquiries can be directed to the corresponding author.

## References

[B1] Bakharev V. D. (1981). Investigation of memory during adaptation to high mountain conditions. *Hum. Physiol.* 7 409–414. 7348227

[B2] Barkaszi I. Takács E. Czigler I. Balázs L. (2016). Extreme environment effects on cognitive functions: a longitudinal study in high altitude in Antarctica. *Front. Hum. Neurosci*. 10:331. 10.3389/fnhum.2016.00331 27445768 PMC4928492

[B3] Berry D. T. McConnell J. W. Phillips B. A. Carswell C. M. Lamb D. G. Prine B. C. (1989). Isocapnic hypoxemia and neuropsychological functioning. *J. Clin. Exp. Neuropsychol*. 11 241–251. 10.1080/01688638908400886 2494222

[B4] Chen J. He S. Y. (2025). Research progress on adaptive changes of the cardiovascular system in high-altitude environments. *Xin Zang Za Zhi* 37 104–109. 10.12125/j.chj.202305010

[B5] Chen X. Y. Ma L. Sun B. L. Liu X. C. Li N. Zhou R. L. (2018). Effects of methamphetamine addiction on working memory. *Chin. J. Clin. Psychol.* 26 427–431. 10.16128/j.cnki.1005-3611.2018.03.002

[B6] Chiu G. Chatterjee D. Johnson R. Freund G. (2010). The impact of acute hypoxia on learning and memory. *Brain Behav. Immunity* 24:S40. 10.1016/j.bbi.2010.07.131

[B7] Erzurum S. C. Ghosh S. Janocha A. J. Xu W. Bauer S. Bryan N. S. (2007). Higher blood flow and circulating NO products offset high-altitude hypoxia among Tibetans. *Proc. Natl. Acad. Sci. U. S. A*. 104 17593–17598. 10.1073/pnas.0707462104 17971439 PMC2077056

[B8] Evans W. O. Witt N. F. (1966). The interaction of high altitude and psychotropic drug action. *Psychopharmacologia* 10 184–188. 10.1007/BF00455979 5982989

[B9] Girardeau G. Lopes-Dos-Santos V. (2021). Brain neural patterns and the memory function of sleep. *Science* 374 560–564. 10.1126/science.abi8370 34709916 PMC7611961

[B10] Guo C. Y. Zhu Y. Ding J. H. Fan S. L. (2003). An ERP study on the relationship between processing levels and memory encoding. *Acta Psychol. Sin.* 2 150–156.

[B11] Hogan A. M. Virues-Ortega J. Botti A. B. Bucks R. Holloway J. W. Rose-Zerilli M. J. (2010). Development of aptitude at altitude. *Dev. Sci*. 13 533–544. 10.1111/j.1467-7687.2009.00909.x 20443973

[B12] Hoit B. D. Dalton N. D. Erzurum S. C. Laskowski D. Strohl K. P. Beall C. M. (2005). Nitric oxide and cardiopulmonary hemodynamics in Tibetan highlanders. *J. Appl. Physiol*. 99 1796–1801. 10.1152/japplphysiol.00205.2005 16024527

[B13] Ito T. A. Larsen J. T. Smith N. K. Cacioppo J. T. (1998). Negative information weighs more heavily on the brain: the negativity bias in evaluative categorizations. *J. Pers. Soc. Psychol*. 75 887–900. 10.1037//0022-3514.75.4.887 9825526

[B14] Kamat S. R. Banerji B. C. (1972). Study of cardiopulmonary function on exposure to high altitude: I. *Am. Rev. Respir. Dis.* 106 404–413. 10.1164/arrd.1972.106.3.414 5080711

[B15] Kamat S. R. Rao T. L. Sarma B. S. Venkataraman C. Raju V. R. (1972). Study of cardiopulmonary function on exposure to high altitude. II. Effects of prolonged stay at 3,500 to 4,000 meters and reversal on return to sea level. *Am. Rev. Respir. Dis*. 106 414–431. 10.1164/arrd.1972.106.3.414 5080712

[B16] Khader P. Knoth K. Burke M. Ranganath C. Bien S. Rösler F. (2007). Topography and dynamics of associative long-term memory retrieval in humans. *J. Cogn. Neurosci*. 19 493–512. 10.1162/jocn.2007.19.3.493 17335397

[B17] Luck S. J. (2005). *An Introduction to the Event-Related Potential Technique.* Cambridge, MA: MIT Press.

[B18] Luks A. M. Hackett P. H. (2022). Medical conditions and high-altitude travel. *N. Engl. J. Med*. 386 364–373. 10.1056/NEJMra2104829 35081281

[B19] Ma H. L. Mo T. Zeng T. A. Wang Y. (2020). [Long-term exposure to high altitude affects spatial working memory in migrants-evidence from time and frequency domain analysis]. *Sheng Li Xue Bao* 72 181–189. 10.13294/j.aps.2020.0012 32328612

[B20] Ma H. L. Zhang X. J. Yang Z. T. (2017). Effects of long-term high-altitude exposure on attentional networks. *Chin. J. High Altitude Med. Biol.* 38 267–272. 10.13452/j.cnki.jqmc.2017.04.009

[B21] Morgan H. M. Klein C. Boehm S. G. Shapiro K. L. Linden D. E. (2008). Working memory load for faces modulates P300, N170, and N250r. *J. Cogn. Neurosci*. 20 989–1002. 10.1162/jocn.2008.20072 18211245 PMC2577178

[B22] Peng D. L. (2023). *General Psychology*, 6th Edn. Beijing: Beijing Normal University Press.

[B23] Richardson C. Hogan A. M. Bucks R. S. Baya A. Virues-Ortega J. Holloway J. W. (2011). Neurophysiological evidence for cognitive and brain functional adaptation in adolescents living at high altitude. *Clin. Neurophysiol*. 122 1726–1734. 10.1016/j.clinph.2011.02.001 21377415

[B24] Ruchkin D. S. Grafman J. Cameron K. Berndt R. S. (2003). Working memory retention systems. *Behav. Brain Sci.* 26 709–728. 10.1017/S0140525X03000165 15377128

[B25] Ruchkin D. S. Johnson R. Grafman J. Canoune H. Ritter W. (1992). Working memory processes. *Cogn. Brain Res.* 1 53–66. 10.1016/0926-6410(92)90005-C 15497435

[B26] Rugg M. D. Mark R. E. Walla P. Schloerscheidt A. M. Birch C. S. Allan K. (1998). Dissociation of the neural correlates of implicit and explicit memory. *Nature* 392 595–598. 10.1038/33396 9560154

[B27] Shang Q. (2013). *Research on Production Efficiency Based on Mental Load.* Hangzhou: Zhejiang University.

[B28] Sheng H. Ding Y. Jiang Q. Zhang L. Wang Y. (2021). Effects of L-cysteine in hypoxic rats. *J. Anhui Med. Univ.* 56 1201–1204. 10.19405/j.cnki.issn1000-1492.2021.08.006

[B29] Simonson T. S. Yang Y. Huff C. D. Yun H. Qin G. Witherspoon D. J. (2010). Genetic evidence for high-altitude adaptation in Tibet. *Science* 329 72–75. 10.1126/science.1189406 20466884

[B30] Song X. Y. Wu W. T. Cao Y. J. Xu W. J. Xie X. P. Sun W. J. (2025). Comparative analysis of pulmonary function in diverse ethnic adults living at low and high altitudes. *Respir. Res*. 27:11. 10.1186/s12931-025-03434-z 41354826 PMC12797799

[B31] Tanner W. P. Swets J. A. (1954). A decision-making theory of visual detection. *Psychol. Rev.* 61 401–409. 10.1037/h0058700 13215690

[B32] Tao G. Ma H. Su Y. (2024). Effects of long-term exposure to high altitude. *Physiol. Behav.* 287:114700. 10.1016/j.physbeh.2024.114700 39332594

[B33] Vogel E. K. Luck S. J. (2000). The visual N1 component as an index of a discrimination process. *Psychophysiology* 37 190–203. 10.1111/1469-8986.3720190 10731769

[B34] Vogel E. K. Machizawa M. G. (2004). Neural activity and memory capacity. *Nature* 428 748–751. 10.1038/nature02447 15085132

[B35] Wang C. Zhang L. Liu Z. Chen Z. Li Y. Fu Y. (2025). Effects of long-term very high-altitude exposure on cardiopulmonary function of healthy adults in plain areas. *Sci. Rep*. 15:24826. 10.1038/s41598-025-07474-9 40640248 PMC12246193

[B36] Wang L. Gui P. Li L. Ku Y. Bodner M. Fan G. (2016). Neural correlates of heat-evoked pain memory in humans. *J. Neurophysiol*. 115 1596–1604. 10.1152/jn.00126.2015 26740529 PMC4808118

[B37] Wang L. Ma S. L. Zhou P. (2023). Effects of high-altitude topography. *J. Public Health Prevent. Med.* 34 99–103. 10.1038/S41598-025-07474-9 40640248 PMC12246193

[B38] Wang T. T. Mo L. Shu S. Y. (2009). Neural mechanisms of memory. *Acta Physiol. Sin.* 61 395–403. 10.3321/j.issn:0371-0874.2009.05.001 19847359

[B39] Ward G. Beaman P. C. (2024). Working memory model. *Q. J. Exp. Psychol.* 78 310–336. 10.1177/17470218241282093 39223965 PMC11783987

[B40] Wesensten N. J. Crowley J. Balkin T. Kamimori G. Iwanyk E. Pearson N. (1993). Effects of simulated high altitude exposure on long-latency event-related brain potentials and performance. *Aviat. Space Environ. Med.* 64 30–36. 8424737

[B41] Wixted J. T. (2020). The forgotten history of signal detection theory. *J. Exp. Psychol. Learn. Mem. Cogn*. 46 201–233. 10.1037/xlm0000732 31246058

[B42] Xiao Y. (2008). *Reverse Picture Superiority Effect.* Chongqing: Southwest University.

[B43] Yan X. Zhang J. Gong Q. Weng X. (2011). Long-term altitude residence and memory. *Brain Cogn.* 77 53–59. 10.1016/j.bandc.2011.06.002 21767899

[B44] Zhang D. He W. Wang T. Luo W. Zhu X. Gu R. (2014). Three stages of emotional word processing: an ERP study with rapid serial visual presentation. *Soc. Cogn. Affect. Neurosci*. 9 1897–1903. 10.1093/scan/nst188 24526185 PMC4249467

[B45] Zhang D. D. Lin Y. Q. Liu Y. Z. Wang T. Luo W. Zhu X. (2019). Encoding and retrieval of emotions. *Acta Psychol. Sin.* 51 36–47. 10.3724/SP.J.1041.2019.00036

[B46] Zhu M. Xu M. Zhang K. Li J. Ma H. Xia G. (2019). Effect of acute exposure to hypobaric hypoxia on learning and memory in adult Sprague-Dawley rats. *Behav. Brain Res*. 367 82–90. 10.1016/j.bbr.2019.03.047 30928461

[B47] Zhuang J. Droma T. Sutton J. R. McCullough R. E. McCullough R. G. Groves B. M. (1993). Autonomic regulation of heart rate response to exercise in Tibetan and Han residents of Lhasa (3,658 m). *J. Appl. Physiol.* 75 1968–1973. 10.1152/jappl.1993.75.5.1968 8307847

